# Accuracy of transcranial magnetic stimulation and a Bayesian latent class model for diagnosis of spinal cord dysfunction in horses

**DOI:** 10.1111/jvim.15699

**Published:** 2020-02-06

**Authors:** Joke Rijckaert, Els Raes, Sebastien Buczinski, Michèle Dumoulin, Piet Deprez, Luc Van Ham, Gunther van Loon, Bart Pardon

**Affiliations:** ^1^ Department of Large Animal Internal Medicine, Faculty of Veterinary Medicine Ghent University Merelbeke Belgium; ^2^ Department of Veterinary medical imaging and small animal orthopedics Ghent University Merelbeke Belgium; ^3^ Département des sciences cliniques, Faculté de Médecine Vétérinaire Université de Montréal, Université de Montréal St‐Hyacinthe Quebec Canada; ^4^ Department of Surgery and Anaesthesiology of Domestic Animals, Faculty of Veterinary Medicine Ghent University Merelbeke Belgium; ^5^ Small Animal Department, Faculty of Veterinary Medicine Ghent University Merelbeke Belgium

**Keywords:** ataxia, cervical radiographs, cervical vertebral malformation, magnetic motor evoked potentials, myelogram

## Abstract

**Background:**

Spinal cord dysfunction/compression and ataxia are common in horses. Presumptive diagnosis is most commonly based on neurological examination and cervical radiography, but the interest into the diagnostic value of transcranial magnetic stimulation (TMS) with recording of magnetic motor evoked potentials has increased. The problem for the evaluation of diagnostic tests for spinal cord dysfunction is the absence of a gold standard in the living animal.

**Objectives:**

To compare diagnostic accuracy of TMS, cervical radiography, and neurological examination.

**Animals:**

One hundred seventy‐four horses admitted at the clinic for neurological examination.

**Methods:**

Retrospective comparison of neurological examination, cervical radiography, and different TMS criteria, using Bayesian latent class modeling to account for the absence of a gold standard.

**Results:**

The Bayesian estimate of the prevalence (95% CI) of spinal cord dysfunction was 58.1 (48.3%‐68.3%). Sensitivity and specificity of neurological examination were 97.6 (91.4%‐99.9%) and 74.7 (61.0%‐96.3%), for radiography they were 43.0 (32.3%‐54.6%) and 77.3 (67.1%‐86.1%), respectively. Transcranial magnetic stimulation reached a sensitivity and specificity of 87.5 (68.2%‐99.2%) and 97.4 (90.4%‐99.9%). For TMS, the highest accuracy was obtained using the minimum latency time for the pelvic limbs (Youden's index = 0.85). In all evaluated models, cervical radiography performed poorest.

**Clinical Relevance:**

Transcranial magnetic stimulation‐magnetic motor evoked potential (TMS‐MMEP) was the best test to diagnose spinal cord disease, the neurological examination was the second best, but the accuracy of cervical radiography was low. Selecting animals based on neurological examination (highest sensitivity) and confirming disease by TMS‐MMEP (highest specificity) would currently be the optimal diagnostic strategy.

**Abbreviations**
C1‐C7: cervical vertebra 1‐7
CI: confidence interval
CT: computed tomography
CVCM: cervical vertebral compressive myelopathy
EDM: equine degenerative myeloencephalopathy
MMEP: magnetic motor evoked potentials
NAD: neuroaxonal dystrophy
Se_NeurEx_: sensitivity of neurological examination
Se_TMS_: sensitivity of transcranial magnetic stimulation
Sp_NeurEx_: specifcity of neurological examination
Sp_TMS_: specifcity of transcranial magnetic stimulation
TMS: transcranial magnetic stimulation

## 1 INTRODUCTION

Spinal ataxia is common in horses. In the United States, equine protozoal myeloencephalitis is an important cause of spinal ataxia, but worldwide, cervical vertebral compressive myelopathy (CVCM) and neuroaxonal dystrophy (NAD)/equine degenerative myeloencephalopathy (EDM) are common diseases.^1^, ^2^ Ataxic horses are often euthanized because they are no longer suitable for riding purposes and a suspected genetic background makes them less desirable for breeding.

Given the important consequences of a definitive diagnosis, the absence of a true gold standard test for CVCM or EDM/NAD in living horses is problematic. Equine degenerative myeloencephalopathy affected horses often have low serum vitamin E concentrations,^3^ but for definitive diagnosis histopathology is required. Cervical vertebral compressive myelopathy can be detected by myelography, computed tomography (CT), CT myelography, and cervical radiography, but all these techniques still have limitations. Computed tomography scans, large enough to visualize C7 are rarely available and do not enable flexion and extension of the neck to evaluate dynamic compression of the spinal cord. With myelography, dynamic spinal cord compression can be visualized, but general anesthesia is required and the sensitivity appears rather low,^4^ especially for the cranial parts of the neck. Cervical radiography might indicate narrowing of the vertebral canal, but the sensitivity (50%) is actually too low for definitive diagnosis.^5^, ^6^, ^7^, ^8^ So, a presumptive diagnosis is often based on the history of the horse and the clinical neurological examination. However, certainly in subtle cases, the agreement between observers is poor and differentiation from orthopedic causes might be challenging.^9^, ^10^

Transcranial magnetic stimulation (TMS) with recording of magnetic motor evoked potentials (MMEP) is a promising additional test for diagnosis of spinal cord dysfunctions in horses.^11^, ^12^, ^13^, ^14^, ^15^, ^16^ A magnetic 70 mm coil is placed on the head of the horse, at the level of the brain, to perform a magnetic stimulation. This induces descending volleys through the spinal cord, evoking a muscle contraction reflected by the MMEP on the electromyography (EMG) machine. On each MMEP, the latency time, the time between stimulation and onset of muscle contraction, can be measured, which is the most reliable variable.^12^, ^13^ In horses, the mean latency time of 4 MMEP is used, instead of the minimal latency time which is used in humans. In normal horses, the mean latency time is short and has a low SD whereas in horses with spinal cord disease, latency time is more variable and clearly prolonged.^14^, ^15^, ^16^ A recent study that compared TMS with histopathology showed that for diagnosis of spinal cord dysfunction, the optimal cutoff values for latency time were 22 ms in the thoracic and 43 ms in the pelvic limbs.^20^ However, these values have not been validated and neurological examination and cervical radiography have not been evaluated accounting for the absence of a true gold standard test. Therefore, the objectives of the present study were to compare the diagnostic accuracy of TMS, cervical radiography, and clinical examination using Bayesian latent class modeling to account for the absence of a gold standard and to determine the optimal diagnostic criterion for spinal cord dysfunction diagnosis by TMS.

## 2 MATERIAL AND METHODS

### 2.1 Study protocol and horses

A retrospective diagnostic test accuracy study was performed. The study population consisted of 174 horses (99 male castrated, 28 intact male, and 47 female), presented between 2008 and 2018 at Ghent University clinic for confirmation or exclusion of a neurological gait abnormality. All horses were evaluated by a neurological examination, TMS, and cervical radiography. On 75 horses, an orthopedic examination was also performed as orthopedic disease was suspected, but the results were not included in the study.

### 2.2 Examination

#### 2.2.1 Neurological examination

Each horse's neurological function was examined by at least 1 of 5 veterinarians of the clinical staff. All examiners had at least 3 years of experience in performing neurological examinations. Neurological examination was conducted using a previously published protocol.^17^, ^18^ The outcome of the neurological examination was summarized in grade of ataxia.^18^ Briefly, grade 0 represented a normal horse. Grade 1 were animals with subtle deficits visible only under special circumstances and not always consistent. Grade 2 corresponded to animals with mild deficits, but visible at all gaits and tests, including walking in a straight line. Grade 3 were horses with moderate deficits visible to any untrained eye from a distance. Grade 4 corresponded to severe deficits with risk of falling easily even if just standing. Recumbent horses, unable to stand, were classified as grade 5 ataxia. For analytic purposes, a binary outcome variable was created grouping horses with grade 0 and 1 as normal (negative test outcome), and horses with grade 2 to 5 as ataxic (positive test outcome).

#### 2.2.2 Cervical radiography

For all horses, lateral radiographs of the cervical vertebrae were made from the occiput to the first thoracic vertebra with a ceiling mounted Phillips X‐Ray tube (80 kW). Output parameters varied from 70 kV/25 mAs for the cranial cervical vertebrae to 90 kV/90 mAs for C7‐T1. A CR system (Agfa DXM) was used with a grid. All radiographs were anonymized and evaluated for any abnormalities by a blinded, board‐certified radiologist. Additionally, the intra‐ and intervertebral sagittal diameter ratios of the vertebral canal were measured at each cervical vertebra as described.^19^ For both ratios, a cutoff value of 0.485 was used to distinguish between a normal and a narrowed vertebral canal indicative for spinal cord compression.^19^

#### 2.2.3 Transcranial magnetic stimulation

For TMS‐magnetic motor evoked potential (MMEP), the procedure described by Nollet et al^12^ was followed. Each horse was sedated with a combination of detomidine (12 μg/kg bodyweight, Domidine, Eurovet Animal Health, Bladel, the Netherlands) and butorphanol (12 μg/kg bodyweight, Dolorex, MSD Animal Health, Boxmeer, the Netherlands). A magnetic stimulator (Magstim 200, The Magstim Company Ltd, Whitland, United Kingdom) and a round 70 mm coil were used to generate a maximal magnetic field of 4 Tesla at the coil surface. The coil was centered over the forehead and maximal stimulus intensity (100%) was applied.^12^ A standard electromyograph (Medelec Sapphire, Medelec Ltd, Surrey, United Kingdom) recorded the muscle responses from the tibialis cranialis and the extensor carpi radialis muscle through intramuscular needle (25 mm monopolar, disposable, insulated, stainless steel needle, TECA Corporation, Pleasantville, New York) or adhesive surface electrodes (Skintact FS50, Skintact, Innsbruck, Austria). These electrode types do not have a significant influence on latency time.^13^ One limb at a time was tested, starting at the left pelvic limb, going to the right pelvic, left thoracic, and finally the right thoracic limb.^13^ For each limb, 4 sequential muscle responses were recorded. For each elicited MMEP, latency time, which is the time interval between the trigger and the first deflection from the baseline, was measured in milliseconds (ms). The cutoff values used for MMEP onset latency time were 21.7 and 42.8 ms for thoracic and pelvic limbs, respectively, based on former performed histopathological research.^20^ A shorter TMS‐MMEP latency time indicates a normal motor conduction through the spinal cord. A latency time equal or longer than the cutoff indicates an abnormal motor function. A binary outcome variable was created based on this cutoff for statistical analysis. All latency time measurements were performed by 1 blinded operator.

### 2.3 Statistics

#### 2.3.1 Definition of outcome tested

As there is currently no gold standard in horses to detect spinal cord disease, Bayesian latent class models were used for accuracy calculation. Bayesian latent class models create their own probabilistic definition of the outcome studied, depending on what the tests actually detect (eg, conductivity or compression by bony structures). In this study, TMS detects conductivity of the spinal cord. Neurologic examination detects ataxia, which is a clinical expression of a sensory disturbance. Cervical radiography visualizes the spinal cord and surrounding bony structures. Hence, the latent variable under consideration is best defined as spinal cord dysfunction, as it is the common factor for all 3 tests.

#### 2.3.2 Model development

15699In order to assess the accuracy of the 3 tests to detect spinal cord dysfunction, we considered a latent class model (1 population 3 tests^21^) allowing for conditional dependence between 2 tests, namely TMS and radiography. We opted for this model because TMS measures the conductivity of the spinal cord, which is disturbed when compressed by bony structures, as measured radiologically. We modeled conditional dependence as previously described.^21^, ^22^ The unknown parameters of interest are the sensitivity and specificity of the 3 diagnostic tests and the prevalence of spinal cord dysfunction in the study population.

The likelihood of the 8 different probabilities of tests results (2^3^ combination for 3 tests) combinations was modeled using the tests characteristics (Se/Sp of NeurEx, RX, and TMS), unknown prevalence of the disease under interest in the studied population (p) and potential conditional dependence of RX and TMS results between horses affected by the latent disease (covDp, covariance in disease positive animals) and nonaffected horses (covDn, covariance in disease negative horses).

1. P(NeurEx+, RX+, TMS+) = p*Se_NeurEx_*(Se_RX_*Se_TMS_+covDp) + (1−p)*(1−Sp_NeurEx_)*((1−Sp_RX_)*(1−Sp_TMS_)+covDn)
2. P (NeurEx+, RX+, TMS−) = p*Se_NeurEx_*(Se_RX_*[1−Se_TMS_]−covDp) + (1−p)*(1−Sp_NeurEx_)*((1−Sp_RX_)*Sp_TMS_−covDn)
3. P(NeurEx+, RX−, TMS+) = p*Se_NeurEx_*((1‐Se_RX_)*Se_TMS_−covDp) + (1−p)*(1−Sp_NeurEx_)*(Sp_RX_*(1−Sp_TMS_)−covDn)
4. P(NeurEx+, RX−, TMS−) = p*Se_NeurEx_*((1−Se_RX_)*(1−Se_TMS_) + covDp) + (1−p)*(1−Sp_NeurEx_)*(Sp_RX_*Sp_TMS_+covDn)
5. P(NeurEx−, RX+, TMS+) = p*(1−Se_NeurEx)_*(Se_RX_*Se_TMS_+covDp) + (1−p)*Sp_NeurEx_*((1−Sp_RX_)*(1−Sp_TMS_)+covDn)
6. P(NeurEx−, RX+, TMS−) = p*(1−Se_NeurEx_)*(Se_RX_*(1−Se_TMS_)−covDp) + (1−p)*Sp_NeurEx_*((1−Sp_RX_)*Sp_TMS_−covDn)
7. P(NeurEx−, RX−, TMS+) = p*(1−Se_NeurEx_)*((1−Se_RX_)*Se_TMS_−covDp) + (1−p)*Sp_NeurEx_*(Sp_RX_*(1−Sp_TMS_)−covDn)
8. P(NeurEx−, RX−, TMS−) = p*(1−Se_NeurEx_)*((1−Se_RX_)*(1−Se_TMS_)+covDp) + (1−p)*Sp_NeurEx_*(Sp_RX_*Sp_TMS_+covDn)

Once the likelihood of the process generating the data observation is described (in our case, a multinomial probability distribution that describes the probability of the 8 tests profiles results), the estimation of posterior densities can be obtained using the Bayes theorem which links the likelihood with the posterior distribution (inference). At this stage, if available, prior information (based on previous studies or on experts opinion) on any of the parameter in the likelihood can be combined to the likelihood to obtain the posterior densities of the different parameters using a Markov Chain Monte‐Carlo algorithm (Gibbs sampling). The sampling is repeated for multiple iterations which will ultimately converge to the posterior distribution.

The prior information is a way to narrow parameter uncertainty when previous scientific information is available. In terms of prevalence and Se/Sp of tests, the priors are modeled using beta distributions that are naturally bound from 0 to 1.^21^ The prior are informative if some values are less probable than others (eg, probability supposed to be higher than a specific value) or uninformative if any value has the same probability of happening (eg, the sensitivity can be anywhere from 0 to 100% with the same probability). The posterior densities after compiling and running the model give an estimate of the probability distribution where the value of the parameter(s) is (are). The Bayesian modeling approach is literally a way to update prior information uncertainty based on the observation of new data combining what is already know to what is not known.

A literature search delivered acceptable prior information for sensitivity and specificity of cervical radiography. For prevalence estimation of spinal cord dysfunction, ataxia, and TMS, information was limited to best guesses by the authors. For TMS, the only available information on diagnostic accuracy was the data set we previously used to identify optimum cutoff values. Hence, we opted to only use prior information on prevalence and sensitivity/specificity of cervical radiography. In all models, we used noninformative priors for Se_TMS_, Sp_TMS_, Se_NeurEx_, and Sp_NeurEx_. A noninformative prior gives an equal probability of any possible value from 0 to 1 which is parametrized as a uniform density from 0 to 1 or a distribution Beta (1, 1). We tested different TMS parameters in this Bayesian framework, comparing with ataxia and radiography.

Because there can be some criticism of the fact that the informative prior elicitation can be a process that could potentially have an impact on posterior density, especially for small data sets, it is recommended to run alternative models with different prior specifications to the main model. This process is called “sensitivity analysis” and is important to see if posterior estimates of alternative models are included in the 95% credibility intervals of the main model.^23^ Assessment of model sensitivity to priors was therefore done by evaluating 3 models. The first model used noninformative priors on prevalence and the 3 tests. In model 2, prior information on prevalence of spinal cord dysfunction was added, and in model 3, prior information on prevalence, sensitivity, and specificity of radiography were added.

#### 2.3.3 Prior distribution determination process

Prior information was derived from available literature and expert opinion. As in the present study population including a lot of horses suspected of a neurological disease, the prevalence of spinal cord disease was estimated at 60% with 95% certainty it would be less than 90% (beta (1.4, 3.1)). The range in which the researchers were 95% confident that the true value of the prevalence was above (or below) was obtained from 2 experts, blinded to each other's guesses. Sensitivity and specificity to detect spinal cord compression on cervical radiography was estimated at 0.50 and 0.70^8^ with 95% certainty it would be more than 0.10 and 0.40, respectively. These values were used to determine the beta distribution parameters of the corresponding prior distribution using a free online beta distribution calculator (epitools, Sergeant, ESG, 2013, AusVet animal Health Services and Australian Biosecurity cooperative Research Centre for Emerging Infectious Diseases) available at http://epitools.ausvet.com.au.

The parameters of interest were determined based on a sample from the posterior distribution using Gibbs sampling with the WinBUGS statistical freeware (version 1.4.3., MRC Biostatistics unit, Cambridge, United Kingdom). Estimation of posterior densities and model assessment was done using recommended techniques.^23^ Each model was assessed after a burn in of 5000 iterations and a total number of 100 000 iterations. The posterior median and 2.5‐97.5 credibility intervals (95% CI) were extracted for each parameter. A total of 3 chains with different initial values was used. Model convergence was checked by visual inspection of density and Gelman‐Rubin plots. Plots of chain autocorrelation were inspected to investigate the need of thinning of the chains.

To determine which TMS criterion is most suitable for spinal cord dysfunction, we evaluated the following TMS criteria in the Bayesian framework, using the 3 models described above each time. The criteria evaluated were: mean latency time of 8 thoracic measurements, mean latency time of 8 pelvic limb measurements, minimal latency time of 8 thoracic measurements, minimal latency time of 8 pelvic limb measurements, minimal of 8 thoracic or minimal of 8 pelvic latency times abnormal or minimal of 8 thoracic and minimum of 8 pelvic latency times abnormal. Each time sensitivity and specificity were determined and to identify the TMS criteria with highest combined sensitivity and specificity, the Youden's index (sensitivity + specificity − 1) was used.

## 3 RESULTS

The age of the horses ranged from 1 to 21 (median 5.5) years and their weight from 230 to 750 (median 555) kg. Most horses (146) were European Warmbloods, 9 were coldblooded types, 4 were Quarter horses, 3 Standardbred, and 1 was Thoroughbred. Eleven horses were presented for prepurchase examination, 58 were suspected to be ataxic, 34 horses showed signs of weakness, 52 presented an atypical lameness, and 19 performed poorly or were reluctant to work.

All latent class models converged. A conditional dependence scenario was used, because the study was underpowered to reject conditional dependence. All parameters were relatively stable across the different models with less than 5% variation compared to the posterior medians.

Estimated prevalence of spinal cord dysfunction varied for the different TMS decision criteria between 43.1 (29.3%‐58.3%) and 60.5 (49.5%‐70.8%). For every decision criterion, the variation between the different models was limited to maximal 5%. In Table 1, the Youden's index for all models is shown. The overall best performing test (Youden's index = 0.85) was TMS‐MMEP using the minimum latency time for the pelvic limbs. Also for 3 other different decision criteria, TMS‐MMEP had the highest Youden's index, indicating it was the best performing single‐diagnostic test. The neurological examination followed on the second place with a maximal index of 0.80. For 5 out of 6 decision criteria, cervical radiography was the poorest test (Youden's index = 0.18‐0.31). The highest sensitivity was found for the neurological examination (0.73‐0.99), whereas TMS‐MMEP was most specific (0.67‐0.97).

**Table 1:** Youden's index (sensitivity + specificity −1) for transcranial magnetic stimulation‐magnetic motor evoked potential (TMS‐MMEP), neurological examination, and cervical radiography, derived from the informed model 3 of for each TMS‐MMEP latency time decision criterion

		TMS‐MMEP	Neurological examination	Cervical radiography
1	Minimum pelvic	**0.85**	0.72	0.20
2	Mean pelvic	**0.81**	0.80	0.18
3	Minimum thoracic OR pelvic	**0.77**	0.63	0.24
4	Minimum thoracic	**0.71**	0.49	0.27
5	Mean thoracic	0.61	**0.80**	0.31
6	Minimum thoracic AND pelvic	0.27	**0.72**	0.62

*Note*: For each decision criterion, the highest values are bolded.

The 2 most valuable TMS‐MMEP decision criteria for practice were the minimal latency time of the pelvic limbs (Table 2) and the mean latency time of the pelvic limbs (Table 3). The highest TMS‐MMEP sensitivity was achieved by using the mean pelvic limb latency time (sensitivity = 0.95) or the minimal latency time of thoracic or pelvic limbs (sensitivity = 0.92). The highest TMS‐MMEP specificity was achieved using the minimal (specificity = 0.97) or mean (specificity = 0.86) pelvic latency time.

**Table 2:** Posterior means and 95% credibility intervals of Bayesian latent class modeling for prevalence (Prev.), sensitivity (Se), and specificity (Sp) of neurological examination (NeurEx), cervical radiographs (RX), and TMS‐MMEP (MMEP) to diagnose spinal cord disease in horses, using the minimum latency times of the pelvic limbs

	Model 1		Model 2		Model 3	
	Prior densities	Posterior densities, median (95% BCI)	Prior densities	Posterior densities, median (95% BCI)	Prior densities	Posterior densities, median (95% BCI)
Se_NeurEx_	Beta (1, 1)	97.6 (91.1‐99.9)	Beta (1, 1)	97.6 (91.4‐99.9)	Beta (1, 1)	97.6 (91.4‐99.9)
Sp_NeurEx_	Beta (1, 1)	76.0 (61.6‐97.5)	Beta (1, 1)	84.8 (61.0‐96.1)	Beta (1, 1)	74.7 (61.0‐96.3)
Sp_RX_	Beta (1, 1)	78.3 (67.4‐87.5)	Beta (1, 1)	78.1 (67.2‐87.3)	Beta (6.3,3.3)	77.3 (67.1‐86.1)
Se_TMS_	Beta (1, 1)	85.9 (67.2‐98.7)	Beta (1, 1)	87.3 (68.4‐99.0)	Beta (1, 1)	87.5 (68.2‐99.2)
Sp_TMS_	Beta (1, 1)	97.4 (90.6‐99.9)	Beta (1, 1)	97.3 (90.4‐99.9)	Beta (1, 1)	97.4 (90.4‐99.9)
Prev.	Beta (1, 1)	49.8 (38.6‐63.8)	Beta (1.4, 3.1)	48.4 (37.6‐61.9)	Beta (1.4, 3.1)	48.3 (37.8‐62.1)
covDp	U (0, a)	−0.0 (−0.06 to 0.04)	U (0, a)	−0.0 (−0.06 to 0.04)	U (0, a)	−0.01 (−0.06 to 0.04)
covDn	U (0, b)	0.0 (−0.01 to 0.03)	U (0, b)	0.0 (−0.01 to 0.03)	U (0, b)	0.010 (−0.02 to 0.03)

*Note*: The prior densities were either noninformative (beta (1, 1)) indicating that all probabilities from 0 to 1 were equally probable or informative. The covariance between the TMS and RX test were parametrized using Dendukuri and Joseph modeling.^21^ The prior distribution of covDp was modeled as a uniform (U) probability bounded between 0 and a = min (Se_RX_, Se_TMS_) − Se_RX_ × Se_TMS_), indicating that all values between these 2 bounds were equally probable. Similarly covDn was modeled as a uniform value between 0 and b = (Sp_RX_, Sp_TMS_) − Sp_RX_ × Sp_TMS_). Model 1: No informative priors. Model 2: Informative prior on prevalence of cervical conductive disturbance (mode 60%; 5th percentile = 10%) corresponding to a beta (1.4, 3.1) distribution. Model 3: Informative priors on prevalence and Se_RX_ (mode 50%; 5th percentile = 10%) and Sp_RX_ (mode 70%; 5th percentile = 40%) corresponding to beta (3.3, 3.3) and beta (6.3, 3.3) distributions.

Abbreviations: BCI, Bayesian credibility intervals; covDn, covariance for negatives; covDp, covariance for positives.

**Table 3:** Posterior means and 95% credibility intervals of Bayesian latent class modeling for prevalence (Prev.), sensitivity (Se), and specificity (Sp) of neurological examination (NeurEx), cervical radiographs (RX), and TMS‐MMEP (MMEP) to diagnose spinal cord disease in horses, using the mean latency times of the pelvic limbs

	Model 1		Model 2		Model 3	
	Prior densities	Posterior densities, median (95% BCI)	Prior densities	Posterior densities, median (95% BCI)	Prior densities	Posterior densities, median (95% BCI)
Se_NeurEx_	Beta (1, 1)	98.3 (91.0‐99.9)	Beta (1, 1)	98.4 (91.6‐99.9)	Beta (1, 1)	98.5 (92.2‐99.9)
Sp_NeurEx_	Beta (1, 1)	82.3 (67.5‐98.4)	Beta (1, 1)	81.4 (67.1‐97.6)	Beta (1, 1)	81.5 (67.2‐97.6)
Se_RX_	Beta (1, 1)	40.9 (30.6‐51.8)	Beta (1, 1)	40.9 (30.7‐51.7)	Beta (3.3,3.3)	41.2 (31.0‐51.7)
Sp_RX_	Beta (1, 1)	77.8 (65.1‐89.0)	Beta (1, 1)	77.2 (64.4‐88.5)	Beta (6.3,3.3)	76.3 (64.6‐86.3)
Se_MMEP_	Beta (1, 1)	94.2 (82.6‐99.7)	Beta (1, 1)	94.7 (83.5‐99.7)	Beta (1, 1)	94.6 (83.2‐99.7)
Sp_MMEP_	Beta (1, 1)	87.3 (75.8‐97.2)	Beta (1, 1)	86.8 (75.0‐96.6)	Beta (1, 1)	86.3 (75.3‐95.1)
Prev.	Beta (1, 1)	59.5 (49.4‐69.9)	Beta (1.4, 3.1)	58.1 (48.3‐68.5)	Beta (1.4, 3.1)	58.1 (48.3‐68.3)
covDp	U (0, a)	0.0 (−0.03 to 0.04)	U (0, a)	0.08 (0.02‐0.15)	U (0, a)	0.0 (−0.03 to 0.04)
covDn	U (0, b)	0.08 (0.01‐0.15)	U (0, b)	0.0 (−0.03 to 0.03)	U (0, b)	0.09 (0.03‐0.15)

*Note*: The prior densities were either noninformative (beta (1, 1)) indicating that all probabilities from 0 to 1 were equally probable or informative. The covariance between the TMS and RX test were parametrized using Dendukuri and Joseph modeling.^21^ The prior distribution of covDp was modeled as a uniform (U) probability bounded between 0 and a = min (Se_RX_, Se_TMS_) − Se_RX_ × Se_TMS_), indicating that all values between these 2 bounds were equally probable. Similarly, covDn was modeled as a uniform value between 0 and b = (Sp_RX_, Sp_TMS_) − Sp_RX_ × Sp_TMS_). Model 1: No informative priors. Model 2: Informative prior on prevalence of cervical conductive disturbance (mode 60%; 5th percentile = 10%) corresponding to a beta (1.4, 3.1) distribution. Model 3: Informative priors on prevalence and Se_RX_ (mode 50%; 5th percentile 10%) and Sp_RX_ (mode 70%; 5th percentile = 40%) corresponding to beta (3.3, 3.3) and beta (6.3, 3.3) distributions.

Abbreviations: BCI, Bayesian credibility intervals; covDn, covariance for negatives; covDp, covariance for positives.

## 4 DISCUSSION

This study brought novelty to equine neurology in 2 ways. Not only was it the first study to evaluate TMS in a large population, it also is the first evaluation of available diagnostic tests for spinal cord dysfunction taking into account the absence of a gold standard. In the present study population, with a high prevalence of neurological dysfunction, mainly associated with spinal cord compression, TMS‐MMEP was the best test to detect spinal cord dysfunction and had the highest specificity. The neurological examination was second best and had the highest sensitivity. The accuracy of cervical radiography, especially the sensitivity (40%‐50%), was poor. In this study, we used a Bayesian latent class approach, which allows accounting for imperfect accuracy of the reference standard test. This methodology is currently the most useful reported strategy in these situations because it is at lower risk of bias than other techniques.^24^ Composite reference standard test is commonly used in retrospective studies after reviewing the whole medical file of the patients. However, this approach has a higher risk of bias compared to the latent class approach.^25^ Interestingly, the models converged well to their Posterior densities and were not sensitive to prior specification. The median posterior densities were all included within the 95% credible intervals of the main model. These observations are characteristics of a reliable, solid model. Also, despite that we anticipated a conditional dependence between TMS and RX, both covariance parameters were not different from 0 because the 95% credibility interval included 0. However, we chose to keep these covariances in our model because the study was not designed to reject a conditional dependence and might lack power to detect small covariances.

The low accuracy of cervical radiography is known^5^, ^6^, ^7^, ^26^ and can be explained by some limitations of the study. First, the study was designed to evaluate the ability of radiography to detect spinal cord dysfunction, of which spinal cord compression is only a part. Spinal cord diseases like equine herpesvirus myeloencephalitis and EDM/NAD, spinal cord compression caused by soft tissue, lateral compression, or thoracic or lumbar lesions will all not be visible on native cervical radiographs, whereas these will cause abnormalities on the neurological examination and possibly also on TMS‐MMEP. Second, enlarged articular process joints can also cause spinal cord compression, but as they are also common in normal horses without neurological deficits,^27^ they were not included in this study. Third, sensitivity will be influenced by the chosen cutoff values. In the present study, the 0.485 cutoff suggested by Hahn et al^19^ was used. In this study, there were no false positives and spinal cord disease was confirmed with histopathology. Earlier, Moore et al^28^ suggested to use a sagittal ratio of 0.52 for C4‐C5 and 0.56 for C7. Logically, using these cutoff values, the sensitivity will increase, but also the rate of false positives will increase. For example for C4, 8 out of 137 horses were considered positive, 3 of them were ataxic, but 5 were normal control horses and thus false positives. By using a lower cutoff, the rate of false positives could be strongly reduced. Fourth, only a single projection was used in our study, whereas a minimum of 2 is recommended for proper image evaluation. Possibly, diagnostic performance of double projections would be better. Overall the results of our study indicate that cervical radiography is the least discriminating test for the diagnosis of spinal cord dysfunction in horses. Moreover, because of the low sensitivity, the question may rise whether cervical radiographs should still be taken for diagnosis of cervical spinal cord dysfunction, especially given exposure of horse, owner, and veterinarian to radiation. Diagnostic imaging remains essential for identification of compressive lesions, but more advanced techniques such as myelography or CT might be better and more informative options.

Concerning TMS‐MMEP, several decision criteria were tested. Similar to human medicine, minimal latency time delivered a higher overall accuracy for TMS‐MMEP than the mean values. However, by using the mean latency time, a higher sensitivity of MMEP could be achieved. So, the choice for minimal or mean latency time might, in the future, vary depending on the purpose of the diagnostic test. For screening purposes, requiring a high sensitivity, mean latency times are the better option, whereas if confirmation of spinal cord disease is wanted, a high specificity is needed making the minimal latency time more suitable. Furthermore, the accuracy was better for pelvic than for thoracic limbs. Decision making based on thoracic limbs alone or if both thoracic and pelvic limb latency times need to be prolonged does not seem interesting.

Concerning the neurological examination, a limitation was that horses with grade 1 were also considered normal in the present study. This decision was based on the fact that certainly in mild cases, the interobserver agreement about the presence of neurological abnormalities might be poor.^9^, ^10^ Therefore, caution is needed when taking decisions based on the clinical examination, especially when signs are subtle^10^ or when orthopedic disease is present. As the study population also included horses suspected of having orthopedic disease and a positive diagnosis of neurological disease might have a serious impact, the authors chose to give the horses with grade 1 ataxia the benefit of the doubt. By considering horses with grade 1 abnormal, the sensitivity of the neurological examination to detect spinal cord dysfunction will increase, but specificity will decrease.

In conclusion, this study showed that TMS‐MMEP, using the minimal or in second place the mean latency time of the pelvic limbs, is the best diagnostic test to diagnose spinal cord dysfunction in a population of horses admitted with suspected ataxia/lameness or purchase control. In our population, spinal cord dysfunction was mainly because of motor dysfunction (spinal cord compression). Transcranial magnetic stimulation‐magnetic motor evoked potential would not detect abnormalities in sensory function, and is therefore only useful in disorders that cause motor deficits. Hence, if the test population would have contained a large proportion of horses with sensory dysfunction, diagnostic performance of the test would have been estimated lower. The neurological examination was the second best diagnostic test and had the highest sensitivity. The accuracy of the cervical radiography was low. Therefore, the authors suggest to screen horses with the neurological examination and to confirm spinal cord dysfunction using TMS‐MMEP. Based on this outcome, decisions can be taken concerning further examinations to find the exact etiology of disease. Because the accuracy of cervical radiography was low, other imaging techniques such as myelography or CT might be a better choice.

## CONFLICT OF INTEREST DECLARATION

Authors declare no conflict of interest.

## OFF‐LABEL ANTIMICROBIAL DECLARATION

Authors declare no off‐label use of antimicrobials.

## INSTITUTIONAL ANIMAL CARE AND USE COMMITTEE (IACUC) OR OTHER APPROVAL DECLARATION

Authors declare no IACUC or other approval was needed.

## HUMAN ETHICS APPROVAL DECLARATION

Authors declare human ethics approval was not needed for this study.
